# Global Lymphatic Filariasis Post-Validation Surveillance Activities in 2025: A Scoping Review

**DOI:** 10.3390/tropicalmed11010028

**Published:** 2026-01-19

**Authors:** Holly Jian, Harriet Lawford, Angus McLure, Colleen Lau, Adam Craig

**Affiliations:** 1 Centre for Clinical Research, Faculty of Health, Medicine and Behavioural Sciences, The University of Queensland, Herston, QLD 4006, Australia; 2 National Centre for Epidemiology and Population Health, The Australia National University, Acton, ACT 2601, Australia

**Keywords:** disease surveillance, neglected tropical diseases, lymphatic filariasis, elephantiasis, elimination

## Abstract

Following World Health Organization (WHO) validation of lymphatic filariasis (LF) elimination as a public health problem, countries are required to implement post-validation surveillance (PVS) to detect potential resurgence and ensure sustained elimination. WHO’s guidelines released in 2025 recommend implementation of at least two of four PVS strategies—targeted surveys, integration into standardised surveys, health facility-based screening, and molecular xenomonitoring (MX) of mosquitoes. This review synthesised global evidence on PVS activities from 2007 to 2025 in the 23 countries and territories validated as having eliminated LF. Studies were identified through PubMed, Scopus, Embase, Web of Science, and the WHO Institutional Repository for Information Sharing (IRIS). Data on publication information, surveillance strategies, priority populations, and operational challenges and enablers were extracted. Narrative synthesis using deductive content analysis was applied. Thirty documents from 17 countries were included. Targeted surveillance and integration of PVS with other health programmes were the most common approaches noted (reported in ten and nine countries, respectively), followed by MX (seven countries) and health facility-based screening (four countries). Surveillance often focused on migrants and previous hotspots, with operational challenges linked to limited funding, workforce, and supply chains. Documents indicated that Sri Lanka, Thailand, China, and South Korea developed sustained PVS through national policies and domestic funding. Findings highlight the need for clear, contextualised guidance to operationalise sustainable PVS in different settings.

## 1. Introduction

Lymphatic filariasis (LF) is a neglected tropical disease caused by three species of thread-like parasitic worms: *Wuchereria bancrofti*, *Brugia malayi*, and *B. timori*. *W. bancrofti* causes 90% of infections worldwide and is spread primarily by *Aedes*, *Anopheles*, and *Culex* genus mosquitoes. The typical vectors for *Brugia* spp. are mosquito species in the genera *Mansonia* and *Aedes* [1,2]. LF is endemic in 72 countries worldwide [3]. Chronic infection can lead to lymphoedema (tissue swelling caused by lymphatic dysfunction), elephantiasis (skin and tissue thickening) of limbs, and scrotal hydrocoele [4]. The resulting disfigurement and disability can lead to physical impairment and social stigmatisation, and subsequent ostracism and loss of employment [5]. Prior to the establishment of the Global Programme for the Elimination of Lymphatic Filariasis (GPELF), chronic LF had an estimated burden of 5.25 million disability-adjusted life years (DALYs) and an estimated economic burden of USD 2.5 billion annually, largely due to disability and loss of employment. In 2019, the burden was estimated to be 1.63 million DALYs [6,7].

The GPELF was launched in 2000 and is one of the largest global public health interventions ever mounted [8]. The programme aims to eliminate LF as a public health problem through multiple rounds of mass drug administration (MDA), as well as managing morbidity and preventing disability (MMDP) among those already infected [8,9]. Regional and national programmes were also established, including the Pacific Programme for the Elimination of Lymphatic Filariasis (PacELF) in 1999 [1], the South-East Asia regional programme to eliminate lymphatic filariasis in 2000 [10], and various national programmes across the World Health Organization (WHO) Regional Office for Africa [11].

The WHO defines elimination of LF as a public health problem as reducing antigen or antibody prevalence to below 1% in both *W. bancrofti*- and *Brugia* spp.-endemic regions, respectively; this represents a threshold at which further transmission is believed to be unlikely even if MDA does not continue [12,13]. The WHO *Road Map for Neglected Tropical Diseases 2021–2030* (the Road Map) aims for all 72 LF-endemic countries to no longer require MDA and to be implementing post-validation surveillance (PVS) or post-MDA surveillance by 2030 [14].

Following elimination as a public health problem, countries are validated by WHO; the validation process requires the preparation and submission of a dossier presenting evidence of sustained reduction in LF nationally [15]. This evidence is based on initial baseline prevalence surveys (known as A/B/C surveys), followed by a transmission assessment survey (TAS1) showing that the prevalence of infection is below the target threshold of 1% in a sample of children under seven years of age, which confirms MDA can be stopped. Subsequent TASs must then show that infection prevalence remains below 1% in children aged under seven years after two years (TAS2) and four years (TAS3) [15]. The Road Map aims for at least 80% of LF-endemic countries (58/72) to have been validated for the elimination of LF as a public health problem by 2030 [16].

The dossier must also include a commitment to and description of proposed post-validation surveillance (PVS) activities [15]. PVS is defined as ongoing, sustained surveillance following elimination, with the primary aim of detecting and preventing resurgence of infection [13]. Due to LF’s long asymptomatic period and its low prevalence in a post-validation context, active case finding is required for PVS [13].

**Figure 1:**
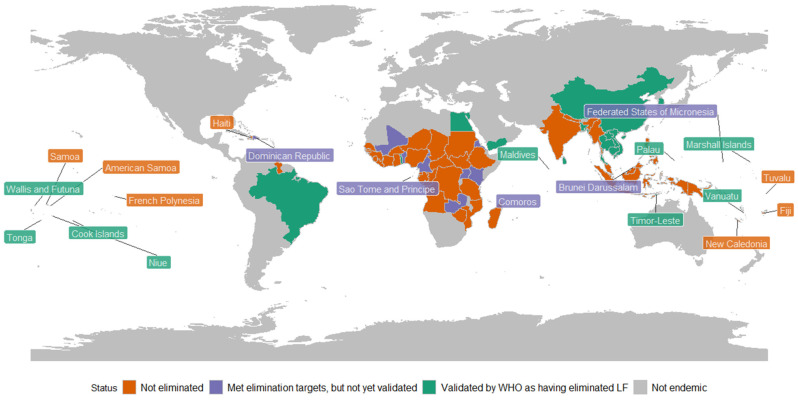
Status of LF elimination as a public health problem in endemic countries, 2025. Data source: World Health Organization [21].

In 2007, China became the first country to be validated by WHO as having eliminated LF as a public health problem. Since then (up to November 2025), 22 additional countries have met the epidemiological threshold, prepared and submitted a dossier, and been validated by WHO as having eliminated LF. Twelve others have completed MDA but are still completing TAS; 39 countries still require MDA [3,9,17]. Figure 1 illustrates countries by LF elimination status, defined as not endemic, not eliminated, having met the epidemiological threshold for elimination (in the process of preparing the validation dossier), and validated by the WHO as having eliminated LF.

WHO guidelines recommend that PVS be continued for at least ten years after validation to monitor for resurgence [13]. Where transmission is ongoing and has not been detected through PVS activities, this may result in re-establishment of transmission, threatening the gains from many years of MDA. Evidence emerging from several Pacific Island countries and territories and Sri Lanka indicates that, even after the criteria for elimination as a public health problem have been met, persistent LF transmission can continue [18,19,20], emphasising the need for sustained PVS. The second edition of the WHO’s guidelines for *Monitoring and epidemiological assessment of mass drug administration in the global programme to eliminate lymphatic filariasis* (released May 2025; hereafter referred to in this document as the WHO guidelines) includes the latest advice on approaches for conducting PVS activities [13]. These guidelines recommend countries implement at least two of the following four PVS strategies: health facility-based screening, integration of PVS into existing population surveys, targeted LF surveillance of high-risk areas or groups, and molecular xenomonitoring (MX) of mosquitoes [13].

The implementation of effective PVS requires a robust understanding of countries’ unique geographic and epidemiological profile, operational context, and challenges and enablers to PVS implementation [22]. Importantly, experiences in one country may inform approaches taken by another, though context, capacity, and need will inevitably differ. Given these challenges, the aims of this scoping review were to identify and synthesise publicly available evidence on PVS activities globally, establishing an evidence base on which future guidelines and strategies may draw. Specifically, the review aimed to

1. Profile publicly documented PVS activities, or lack thereof, in countries previously endemic for LF.
2. Examine documented barriers and facilitators to the implementation of PVS strategies and how these vary by context.
3. Compare alignment of PVS activities with recently released recommendations in the WHO’s *Monitoring and epidemiological assessment of mass drug administration in the global programme to eliminate lymphatic filariasis* guidelines [13].
4. Identify knowledge gaps in PVS implementation methods that may be addressed through further operational research.

## 2. Methods

The methods for this study have been registered on PROSPERO (registration ID: CRD42024618436).

### 2.1. Identifying Studies

This study was conducted in line with the Preferred Reporting Items for Systematic reviews and Meta-Analyses (PRISMA) guidelines Extension for Scoping Reviews (PRISMA-ScR) [23]. The Population, Intervention/Exposure, Comparator, Outcome, and Time (PICOT) framework was used to develop the search strategy, presented in Table 1. As this review aimed to scope and critique the PVS strategies being employed across several countries, we determined that identifying a Comparator was not required.

PubMed, Scopus, Embase, and Web of Science were used to search for peer-reviewed journal articles, and the WHO Institutional Repository for Information Sharing (IRIS) database was used to search for PVS-related grey literature reports published by WHO.

A search strategy using Medical Subject Heading (MesH) search terms, free-text keywords, and Boolean operators were developed. Supplementary Materials File S1 presents the search terms used across each literature database.

### 2.2. Data Management, Study Screening, and Selection

Data management, study screening, and selection were performed with the aid of Covidence software (https://www.covidence.org/ (accessed on 2 September 2025)). Search results from the selected databases were imported into Covidence and duplicates removed.

Documents were included if they met all of the following criteria:

- Described PVS activities conducted between 1 January 2007 and 30 July 2025 (inclusive) in a country or territory that has been validated by WHO as having eliminated LF as a public health problem;
- Were original pieces of research, activity reports, protocols, or WHO grey literature describing population-level surveillance;
- Were published between 1 January 2007 and 30 July 2025 (inclusive).

Documents were excluded if they met either of the following criteria:

- Described surveillance activities that occurred before validation by WHO of elimination of LF;
- Did not describe population-level surveillance activities (such as diagnostic validation and modelling studies, letters, editorials, and commentary articles).

Two researchers (HJ and HL) independently screened the title and abstracts of included records using the above criteria. Where disagreement in screening assessments occurred, HJ and HL discussed and aimed to find consensus; if required, a third reviewer (AC) was consulted. If resolution was not found, a conservative approach was taken and the article included for full text review. Following title and abstract screening, HJ conducted full text screening. Screening decisions were documented using a PRISMA flow diagram.

### 2.3. Data Extraction

Data were extracted into an Excel spreadsheet developed by the research team. Data were extracted for four categories:

1. Publication information: year of publication, country of study setting, and publication funding sources;
2. LF epidemiology: causative pathogen (*W. bancrofti* or *Brugia* spp.), year in which LF was validated as eliminated, and targeted high-risk or priority populations (e.g., historic hotspots);
3. PVS activities: years in which PVS activities were conducted, type of surveillance activities conducted, frequency of activities, sampling design (targeted or population-representative), surveillance type (active, passive, or sentinel), type of testing (antigen, antibody, microfilaria, and MX/PCR of mosquito samples), and other activities (including risk reduction and vector management);
4. Operational challenges or enablers: PVS activity funding sources; staffing and resources; technical capacity; and social, political, or economic drivers of PVS programme implementation.

### 2.4. Analysis

A deductive content analysis of the extracted data was conducted using the method developed by Elo and Kyngas (2008) [24]. A deductive approach was selected due to its applicability in testing existing models in different contexts [24]. In this case, this approach was used to examine the application of PVS strategies recommended by WHO in 2025 across countries endemic for LF [13]. Deductive coding was conducted using a constrained categorisation matrix using the data extraction fields described above, with a focus on identifying emerging themes relating to alignment with WHO guidelines, operational challenges, and enablers [24]. The analysis sought to capture information about PVS activities by country and challenges or enablers to their implementation. Additionally, the analysis also examined the alignment of documented efforts with WHO guidelines, specifically the number of activities implemented and whether they were sustained, that is, repeated PVS activities implemented over multiple years following validation, as opposed to single or short-term efforts. Furthermore, the analysis sought to contrast approaches used by countries and extracted learnings that can be translated across settings.

### 2.5. Ethical Considerations and Funding

This review did not require ethical approval as it uses published, publicly available data. This work was supported by the Operational Research and Decision Support for Infectious Diseases (ODeSI) programme, which is funded by The University of Queensland’s Health Research Accelerator (HERA) initiative (2021–2028).

## 3. Results

**Figure 2:**
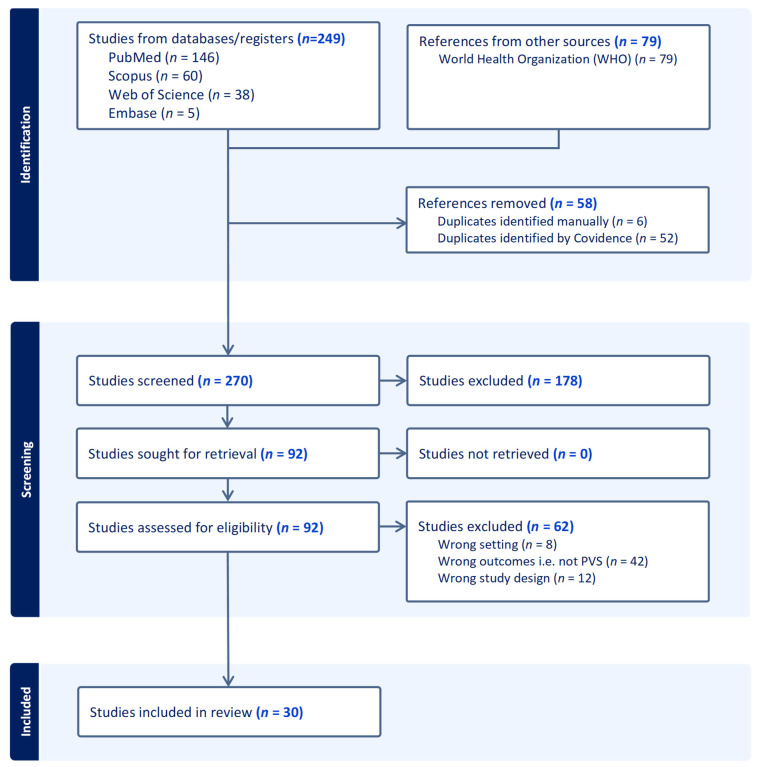
PRISMA diagram showing study selection process.

A total of 328 documents were identified, of which 58 were duplicates. Of the 270 unique documents that remained, 30 met the inclusion criteria (summarised in Table 2). Figure 2 outlines the process of document identification and selection.

The majority of documents (22/30; 73%) were peer-reviewed journal articles and the remainder (*n* = 8) were reports published by the WHO. The documents were published from 2009 to 2025.

Data were reported on PVS activities in 17 countries: Bangladesh [35], Cambodia [34], China [25,26,27,28,29,30,31], Egypt [36], Kiribati [51], Malawi [53], Maldives [36,37,38], Niue [39], Palau [49], South Korea [28,31,32,33], Sri Lanka [19,34,35,36,37,38,40,41,42,43], Thailand [35,36,37,38,44,45], Togo [46,47], Tonga [48], Vanuatu [34], Wallis and Futuna [50], and Yemen [52]. More than half of the countries (53%) for which PVS activities were reported were from the Western Pacific Region (WPR) of the WHO.

### 3.1. Lymphatic Filariasis Elimination

The years in which countries eliminated LF ranged from 2007 to 2023. Almost all documents (29/30, 97%) described activities relating to the surveillance of *W. bancrofti* in all 17 countries represented in the study. A total of 18 of 39 documents (60%) described activities relating to *Brugia* spp. in Sri Lanka, China, South Korea, and Thailand.

The average time between elimination and the first reporting of PVS activities was three years (range 0–9 years). This differed by publication type, with WHO reports published on average two years (range 0–5 years) after validation of elimination, while journal articles were available on average after a six-year delay (range 1–10 years).

### 3.2. Post-Validation Surveillance Activities

The PVS strategies described in the literature are summarised below in Table 3 by country. These include strategies recommended in the 2025 WHO guidelines for PVS as well as other approaches, including vector surveys (monitoring of mosquitos without further dissection or polymerase chain reaction (PCR) testing for filarial DNA) and animal reservoir surveys of dogs and cats (reservoirs for *Brugia* spp.) [54].

### 3.3. WHO-Recommended PVS Strategies

Of the WHO-recommended strategies, targeted surveillance [19,25,26,28,29,31,34,35,37,38,41,42,43,44,45,46,48,49,50,55] and integration of PVS into existing population surveys [34,35,37,38,39,51,52] were the most commonly implemented, described in ten and nine countries, respectively. These were followed by MX (seven countries) [34,35,37,38,39,51,52,55] and health facility-based screening (four countries) [26,28,31,32,33,34,35,37,38,43,44,46,47,50]. However, a combination of the WHO-recommended strategies was described in more than half (nine) of the countries. The most common combination reported was targeted surveillance and MX, described in six countries [26,28,31,34,35,37,38,43,44,46].

Single PVS strategies were described in five countries: Malawi, Egypt, Kiribati, Niue, and Vanuatu. Kiribati, Niue, and Vanuatu reported integrating LF surveillance into other existing population-based surveillance programmes, though the specific programmes were not described in the literature [34,39,51]. Malawi and Egypt reported MMDP programmes but did not specify whether surveillance activities were conducted [53,55].

Documents described activities in nine countries that targeted ‘hotspots’; these were higher-risk areas that were generally formerly endemic regions with higher baseline LF prevalence [19,25,26,28,31,32,34,35,38,41,42,43,44,47,48,50]. Nine countries conducted targeted surveys of migrants from endemic countries [28,29,30,33,35,37,38,39,44,45,46,49,55]. LF surveillance was integrated into surveys for non-communicable diseases (NCDs) (such as the WHO STEPwise survey in Niue [39]), other NTDs such as trachoma, and vaccine-preventable diseases such as tetanus [34]. Additionally, MX for LF was integrated into dengue surveillance programmes in Sri Lanka [38].

Tonga, China, Bangladesh, and Wallis and Futuna employed health facility-based testing. LF testing was integrated into routine checkups at a diabetes clinic in Tonga [48]; in Wallis and Futuna, the diagnosis of patients with lymphoedema and hypereosinophilia prompted a review of medical records and subsequent survey of children under 18 on the island of Futuna [50]. Other passive health facility-based surveillance was noted to have been conducted in China and Bangladesh, though the literature did not describe specific activities [27,35].

MX was described in six countries where it was primarily used to monitor high-risk areas such as previously endemic provinces with historically high LF prevalence. As noted above, MX was not used as a standalone surveillance method and was generally used in conjunction with targeted surveys in high-risk areas [26,28,31,34,35,37,38,43,44,46].

### 3.4. Other Surveillance Strategies

Six countries used surveillance methods that were not one of the four recommended strategies in the 2025 WHO guidelines. One document from China described the presentation of a symptomatic case triggering reactive case finding in their home district/village [25]. Additionally, animal reservoir (cat and dog) surveys were conducted in *Brugia*-endemic regions in Sri Lanka and Thailand [35,37,41,42,44]. Yemen and the Maldives conducted vector surveillance but did not conduct MX, noting constraints to PCR capabilities [37,52]. Wallis and Futuna identified that patients presenting with lymphoedema also had marked hypereosinophilia; following a review of medical records that identified that the prevalence of hypereosinophilia was three times higher in Futuna than in Wallis, a survey of children under 18 on Futuna was triggered, which identified evidence of ongoing LF transmission [50].

### 3.5. Diagnostic Testing Methods

A mix of diagnostic tests were used in PVS testing, with most countries using a combination of testing methods to validate their findings. The most commonly described was antigen testing using Alere Filariasis Test Strips (FTSs), reported by ten countries in *W. bancrofti*-endemic regions [19,28,31,37,38,39,43,44,46,48,49,50,56]. Rapid antibody tests were used in six countries in *Brugia* spp.-endemic regions [19,26,28,31,34,39,41,42,43,44]. Microfilaria (Mf) testing and MX were used to supplement these testing methods in eight [28,37,38,39,41,44,46,48,49,50,56] and five countries, respectively [26,28,31,32,34,37,38,41,43,47]. Most countries that used antigen testing (with the exception of the Maldives) reported conducting follow-up Mf testing [28,37,39,44,46,48,49,50,56].

### 3.6. Frequency of PVS

WHO guidelines recommend that PVS should continue for ten years after validation of LF’s elimination and that data from PVS activities should be submitted annually to WHO; however, the time when PVS should commence, its frequency, or methods are not stipulated [15]. Nine countries conducted one-off activities only (PVS activities that occurred over a single instance or over a short, defined period, rather than as part of an ongoing programme): Malawi [53], Togo [46,47], Yemen [52,55], Kiribati [51], Niue [39], Palau [49], Tonga [48], Wallis and Futuna [50], and Vanuatu [34]. Sustained surveillance (repeated PVS activities implemented over multiple years) after validation of elimination was reported in six countries: the Maldives [35,37,38,55], Sri Lanka [35,37,38,40], Thailand [35,37,38,44,45,55], Cambodia [34], China [25,26,27,28,29,30,31], and South Korea [33]. The strategies used for sustained surveillance were reported as targeted surveys in formerly endemic areas [25,26,28,29,31,35,36,37,38,44,45], migrant screening [28,29,30,33,35,36,37,38,44,45], and MX/vector surveillance or animal reservoir screening [26,31,33,35,37,38,44].

However, the identification of sustained or one-off surveillance should be interpreted cautiously: for example, Togo implemented multiple PVS approaches across three years, which does not constitute sustained surveillance for any single method but is reflective of continued, repeated PVS efforts [46,47]. Similarly, for countries like Tonga, where PVS was only conducted in 2024 and findings published in 2025, it is too early to determine future activities [48]. In addition, the frequency and continuity of PVS are difficult to assess from the published literature alone, as the academic and grey literature are incomplete and often lack detail on timing and repetition of activities or on the future plans of national programmes.

### 3.7. Health System Constraints and Enablers

Eight countries cited financing constraints as a major barrier to PVS implementation, raising concerns about programme sustainability [34,35,37,38,47,48,53]. For example, a 2017 WHO report noted that in Cambodia and Vanuatu, there was no national or international funding for PVS, raising concerns about long-term sustainability due to reliance on external donors [34]. Budget reductions were also described in Thailand, which has conducted sustained PVS since LF’s elimination in 2016; however, consistent funding from both the Thai national government and the WHO were also noted [35,37,38].

Issues relating to health workforce were reported in seven countries [34,35,37,38,40,47,53]. Countries reported significant limitations to technical capacity, such as limited access to laboratories capable of conducting enzyme-linked immunosorbent assay (ELISA) and PCR testing required for LF antibody detection and MX, respectively [34,37]. Shortages of specialist staff can particularly affect MX implementation, due to the requirement for trained entomologists [47]. High staff turnover also affected the quality of MMDP services [53].

Six countries faced challenges in the timely procurement of rapid diagnostic tests (RDTs) and ELISA and PCR testing supplies [34,35,37,38,47]. RDTs were generally not readily available in countries and had to be procured from the WHO; this particularly impacted small island nations, as WHO processes are challenging for the procurement of small quantities [35,37].

Documents from China, Sri Lanka, Thailand, and Tonga commented on the absence or ambiguity of PVS guidelines, including a lack of recommendations on the frequency or duration of PVS and the thresholds for determining sustained elimination [31,34,35,42,43,48]. These documents were published between 2009 and 2025, noting that the latest guidelines on PVS were published in May 2025.

Six countries described enabling factors that strengthened programme sustainability. Notably, Sri Lanka, South Korea, the Maldives, China, and Thailand included LF and other NTDs in national health policies and strategies [25,27,33,35,37]. Other activities included regular education of health professionals on LF surveillance and treatment [33,35,37], the development of databases to monitor LF [19,27,33,35,37], the building of specialist facilities for MMDP [26], and community health education [26,35]. The above health system constraints and enablers are summarised below in Table 4.

### 3.8. Knowledge Gaps

Of the 23 countries that have eliminated LF as a public health problem, there was no literature found on PVS activities for six (26%) countries: the Cook Islands (which was validated as having eliminated LF in 2016), the Republic of the Marshall Islands (in 2017), Vietnam (2018), Lao People’s Democratic Republic (2023), Brazil (2024), and Timor-Leste (2024) [55,57]. As noted above, gaps in the published literature alone may not reflect all PVS activities conducted by countries or future plans of national programmes.

We also noted that in some instances, the nature of PVS activities was reported ambiguously; for example, the specific activities constituting China and Bangladesh’s passive health facility-based surveillance was not described [27,35]. Similarly, LF surveillance in Kiribati, Niue, and Vanuatu was reported to have been integrated into other existing population-based surveillance programmes; however, the nature and frequency of these activities were not specified [34,39,51]. Lastly, two countries (Malawi and Egypt) reported MMDP programmes but did not specify whether other surveillance activities to detect and prevent LF further transmission were conducted [53,55].

## 4. Discussion

This review synthesised 30 documents reporting PVS activities in 17 of the 23 countries and territories validated by WHO as having eliminated LF. An overarching theme in our extracted documents was the tension between constraints to establishing PVS programmes and the need for active case finding due to LF’s long asymptomatic incubation period and low prevalence in a post-validation context.

Of the new WHO-recommended strategies released in 2025 (at the end of this review’s study period) in the guidelines for *Monitoring and epidemiological assessment of mass drug administration in the global programme to eliminate lymphatic filariasis* [13], the literature most commonly reported implementation of targeted surveys focusing on migrants or previous hotspots, in combination with MX or vector surveillance. These approaches were already being implemented before the release of the guidelines, suggesting that countries were pragmatically balancing epidemiological need with resource availability and opportunity. Targeted surveillance was also considered cost-effective, as it concentrates efforts on higher-risk areas and populations most likely to require more intensive follow-up [58,59,60].

While integration of PVS into existing health programmes and health facility-based screening was also described, single-disease, siloed approaches to PVS were reported in several countries included in our study. In the context of constrained resources, this suggests that integration of LF surveillance may be an underutilised opportunity to address the WHO requirement to sustain PVS for ten years. A qualitative study conducted by Craig et al. found that, among Pacific Island country and territory representatives, integration was seen to be well-suited to small island contexts due to their effective use of limited resources and their reduction of the burden on surveyed communities; opportunities identified for integration included other seroprevalence surveys (e.g., to monitor vaccine-preventable diseases) and screening programmes for NCDs, as well as implementing enhanced surveillance through investigating eosinophilia cases identified through routine blood testing [50,61].

However, some documents reinforced the importance of considering each country’s epidemiological context when selecting programmes for integration. In Togo, for example, where the primary risk of LF resurgence stems from cross-border reintroduction, passive laboratory-based surveillance was found to be less effective than targeted surveys conducted in border regions with large migrant populations [46]. Similarly, in Tonga, targeted surveys in remote outer islands identified high-prevalence clusters, whereas no positive cases were detected through screening at a diabetes clinic in the capital, an urban setting less likely to capture individuals from remote communities [48]. These findings underscore the importance of context when designing PVS strategies, particularly where transmission risk is localised or linked to specific populations.

The most commonly reported challenges to PVS implementation were financing constraints [34,35,37,38,47,53], limited technical capacity [34,37,40,47,53], and supply chain issues [34,35,37,38,47]. These were particularly notable among reports related to small island nations and low-income settings [34,35,37,38,47,53], where reliance on external donors’ support and limited laboratory infrastructure hindered sustained surveillance. Addressing these challenges requires strengthening health system capacity, through assessing financing models, improving access to technical support and the availability of essential equipment. Development partners, including donors, are well-positioned to assist countries to overcome these barriers in the short-to-medium term. We also noted that ad hoc approaches do little to strengthen health systems; examining the approach of countries where PVS has been embedded in national health strategies and supported by domestic financing (namely Sri Lanka, the Republic of Korea, China, and Thailand) will provide valuable insights into how surveillance can be designed for long-term sustainability.

Financing constraints likely underpinned other findings we observed across countries, many of which were low- and middle-income and/or had small populations and workforces. For example, in more than half of the countries represented in our review, we only identified evidence for one-off PVS activities; the WHO recommends that PVS should continue for at least ten years, though it does not specify the frequency of interventions [13]. Financing constraints may also account for only a single PVS strategy being described in some countries; while the latest PVS guidelines recommend that at least two PVS activities are used, we noted that this review describes activities that largely take place prior to the guidelines’ release [13]. In settings where resources are stretched, programme managers may prioritise approaches that are feasible within existing budgets or acceptable to funders, even if these do not fully align with international guidance; for example, the literature reported only MMDP activities in Malawi and Egypt to manage the existing burden of chronic disease, with no description of PVS activities to detect and prevent further transmission. These findings highlight the need for sustainable financing mechanisms that are cognisant of contextual challenges to support robust PVS.

Conversely, Sri Lanka, South Korea, China, and Thailand stood out as countries reporting robust and sustained PVS systems, utilising a combination of surveillance strategies. In these four countries, efforts to build governance and programme sustainability were evident, notably through the inclusion of LF and other NTDs in national budgets and health strategies, and a commitment to ongoing implementation [25,33,35,37,44]. These examples suggest that institutional commitment and governance structures are critical enablers of long-term surveillance.

It is important to note that the integration of LF into national health agendas often depends on the availability of clear international guidance. Multiple publications reported difficulties stemming from the absence or ambiguity of PVS guidelines [31,34,35,42,43,48]. Most countries were validated as having eliminated LF as a public health problem between 2016 and 2018; WHO guidelines on specific PVS strategies to use were published in 2025, nearly a decade into many countries’ post-validation phases [13]. Timely guidance is essential to provide a framework for countries to refer to when developing national surveillance strategies.

The majority of PVS activities documented in this review were implemented prior to the release of the 2025 WHO guidelines. While most activities broadly aligned with the four recommended strategies, more than a third of the included countries reported additional approaches not specified in the guidelines, such as animal reservoir surveys. These adaptations reflect pragmatic responses to local epidemiological and operational contexts. However, the absence of guidance on PVS timing and longevity, in combination with lack of funding, has likely contributed to considerable variation in programme sustainment; some countries conducted one-off surveys, while others implemented multi-year programmes. Additionally, only one type of surveillance was recorded in the literature in five countries, while the 2025 WHO guidelines recommend the implementation of at least two. This ambiguity underscores the need for clearer and timely recommendations on PVS programme design to ensure consistency and comparability across settings.

Now that the 2025 PVS guidelines are available, the next critical steps will involve translating these recommendations into actionable strategies fit for each countries’ unique context. This may involve supporting countries to assess epidemiological information, consider context and resource availability, and design appropriate PVS strategies. To ensure community and political support to drive PVS, continued advocacy will be required to stakeholders including policymakers, development agencies, and other international partners. Additionally, the forthcoming WHO *Integrated Surveillance Planning Toolkit for Neglected Tropical Diseases in Post-Validation or Verification Settings* will provide practical guidance to help countries design integrated, risk-based surveillance strategies [62]. Leveraging such tools can support evidence-based planning, facilitate resource mobilisation, and promote integration with existing health systems, reducing costs and operational burden while strengthening sustainability.

A key limitation of our study is that it relied on publicly accessible documented evidence of PVS, and that grey literature searches were limited to the WHO IRIS database. In all likelihood, some activities have been conducted but are yet to be reported; this limits the insights that were able to be drawn, particularly about the longevity of PVS activities. Conversely, the absence of the literature about PVS from six countries that have eliminated LF as a public health problem raises the question as to whether PVS has been conducted. This limits opportunities for cross-country learning and highlights the need for operational research and reflective practice to inform future surveillance strategies, particularly as countries interpret and implement the 2025 WHO PVS guidelines.

## 5. Conclusions

Our review demonstrates that LF PVS is implemented with considerable variation across countries and territories, shaped by differences in epidemiological risk, health system capacity, and resource availability. Targeted surveys and MX were the most frequently reported PVS strategies used, while integration with existing standardised population surveys and health facility-based screening were less commonly reported. Financing and resource constraints underpinned many challenges to PVS implementation, affecting the scope and longevity of surveillance programmes.

Countries with more sustained and integrated approaches to PVS demonstrate how institutional commitment and governance structures are critical enablers of sustained surveillance. The findings also highlight the importance of timely international guidance, particularly given the lag between LF validation and the release of WHO’s 2025 PVS recommendations. Strengthening national ownership, improving integration with existing health systems, and ensuring access to technical and financial resources will be essential to maintaining LF elimination and preventing resurgence.

## Figures and Tables

**Table 1:** PICOT components.

PICOT	Component	Search Term	Add with
Population	Countries that have been validated as having eliminated LF by WHO	“Bangladesh” [MesH] OR Bangladesh * OR “Cambodia” [MesH] OR Cambodia * OR China [MesH] OR Cook Island * OR “Egypt” [MesH] OR Egypt * OR Kiribati * OR “i-Kiribati” OR “Laos” [MesH] OR “Laos” OR “Malawi” [MesH] OR Malawi * OR Maldiv * OR Marshall Island * OR Niue * OR Palau * OR “Republic of Korea” [MesH] OR “South Korea” OR “Sri Lanka” [MesH] OR Sri Lanka * OR “Thailand” [MesH] OR Thai * OR “Togo” [MesH] OR Togo * OR Tonga * OR Vanuatu * OR “Vietnam” [MesH] OR Vietnam * OR “Yemen” [MesH] OR Yemen * OR “Wallis and Futuna” OR Wallis * OR Futuna * OR “Brazil” [MesH] OR Brazil * OR “Timor-Leste” [MesH] OR Timor *	AND
Exposure or intervention	Lymphatic filariasis	“Elephantiasis, Filarial” [MesH] OR “lymphatic filariasis” OR elephantias * OR filaria * OR “filarial elephantiasis” OR filarial lympho *dema OR “*Wuchereria bancrofti*” OR “*Brugia malayi*” OR “*Brugia timori*” OR *Bancrofti* * OR *Brugia* *	AND
Outcome	Post-validation surveillance	“post-elimination” OR “post-validation” OR elimination OR validation OR “Sentinel Surveillance” [MesH] OR “sentinel surveillance” OR “Public Health Surveillance” [MesH] OR “public health surveillance” OR “Population Surveillance” [MesH] OR “population surveillance” OR “Monitoring, Physiologic” [MesH] OR “physiologic monitoring” OR “Epidemiological Monitoring” [MesH] OR “Mass Screening” [MesH] OR “mass screening”	AND
Comparator	N/A	N/A	
Time	January 2007–June 2025 (inclusive)	January 2007–June 2025 (inclusive)	AND

*—wildcard search term used to indicate matches to any number or sequence of characters. N/A—not applicable.

**Table 2:** Summary of documents reporting PVS activities.

Country	Year LF Eliminated	WHO Region	Causative Pathogen	Reference	Year Published	Activities	Priority Populations *	Years Conducted
China	2007	WPR	*W. bancrofti*, *B. malayi*	Sun et al. [25]	2020	Targeted surveys	Historic hotspots	2008
			*W. bancrofti*, *B. malayi*	Huang et al. [26]	2020	Targeted surveys, MX, MMDP	Historic hotspots	1982–1992 ^
			*W. bancrofti*, *B. malayi*	Qian et al. [27]	2019	Health facility-based passive surveillance	Population-wide	1980 onwards ^
			*W. bancrofti*, *B. malayi*	WHO [28]	2018	Targeted surveys, passive surveillance	Migrants from endemic countries	2007 onwards
			*W. bancrofti*, *B. malayi*	Yang et al. [29]	2014	Targeted surveys, MMDP (incl. the establishment of new facilities)	Migrants from endemic countries	2007 onwards
			*W. bancrofti*, *B. malayi*	Sudomo et al. [30]	2010	MMDP	Migrants from endemic countries	2007 onwards
			*W. bancrofti*, *B. malayi*	WHO [31]	2009	Targeted surveys, MX, MMDP	Areas known to have weak surveillance systems and/or symptomatic cases, migrants from endemic countries	2007 onwards
South Korea	2008	WPR	*B. malayi*	Riches et al. [32]	2020	MX	Historic hotspots	2011
			*W. bancrofti*, *B. malayi*	Bahk et al. [33]	2018	Sentinel surveillance	Migrants from endemic countries	Unspecified
			*B. malayi*	WHO [28]	2018	Targeted surveys, MX	Historic hotspots	School surveys 2009–2011; MX 2008 onwards
			*W. bancrofti*, *B. malayi*	WHO [31]	2009	Targeted surveys, MX	Historic hotspots	2009
Cambodia	2016	WPR	*W. bancrofti*	WHO [34]	2017	Integrated surveillance	Historic hotspots	Unspecified
Maldives	2016	SEAR	*W. bancrofti*	WHO [35]	2025	School-based surveys, targeted surveys	Migrants from endemic countries	2016 onwards
			*W. bancrofti*	WHO [36]	2024	Targeted surveys	Historic hotspots, migrants from endemic countries	2016 onwards
			*W. bancrofti*	WHO [37]	2023	Targeted surveys, integrated surveillance, integrated MX, MMDP	Migrants from endemic countries	2016 onwards
			*W. bancrofti*	WHO [38]	2020	Targeted surveys, integrated MX	Migrants from endemic countries	2016 onwards
Niue	2016	WPR	*W. bancrofti*	Craig et al. [39]	2025	Integration into STEPs	Migrants from endemic countries	2024
Sri Lanka	2016	SEAR	*W. bancrofti*, *B. malayi*	WHO [35]	2025	Targeted surveys, MX	Unspecified	2017 onwards
			*W. bancrofti*, *B. malayi*	WHO [36]	2024	Targeted surveys	Historic hotspots, migrants from endemic countries	Unspecified
			*W. bancrofti*, *B. malayi*	Gunaratna et al. [40]	2024	MMDP	Unspecified	2016 onwards
			*W. bancrofti*, *B. malayi*	WHO [37]	2023	Targeted surveys, MX	Migrants from endemic countries	2016 onwards
			*W. bancrofti*, *B. malayi*	Mallawarachchi et al. [41]	2021	Targeted surveys, MX, cat and dog serosurveys	Historic hotspots	After 2018 (years unspecified)
			*B. malayi*	WHO [38]	2020	Targeted surveys, integrated MX, MMDP	Migrants from endemic countries	Unspecified
			*W. bancrofti*, *B. malayi*	Rahman et al. [19]	2019	Targeted surveys	Historic hotspots	2018
			*B. malayi*	Mallawarachchi et al. [42]	2018	Targeted surveys, dog serosurveys	Historic hotspots	2016–2017
			*W. bancrofti*, *B. malayi*	WHO [34]	2017	Targeted surveys	Historic hotspots	2017 onwards
			*W. bancrofti*	Rao et al. [43]	2017	Targeted surveys, MX	Historic hotspots	2015–2017
Vanuatu	2016	WPR	*W. bancrofti*	WHO [34]	2017	Integrated surveillance	Unspecified	Unspecified
Thailand	2017	WPR	*W. bancrofti*, *B. malayi*	WHO [35]	2025	Targeted surveys, cat serosurveys, integration with NCD screening	Migrants from endemic countries	2017 onwards
			*W. bancrofti*, *B. malayi*	WHO [36]	2024	Targeted surveys	Historic hotspots, migrants from endemic countries	2017 onwards
			*W. bancrofti*, *B. malayi*	Meetham et al. [44]	2023	Targeted surveys, cat serosurveys	Historic hotspots, migrants from endemic countries	2017 onwards
			*W. bancrofti*, *B. malayi*	WHO [37]	2023	Targeted surveys, MX, cat serosurveys	Migrants from endemic countries	2017 onwards
			*W. bancrofti*, *B. malayi*	Bizhani et al. [45]	2021	Targeted surveys	Migrants from endemic countries	2017 onwards
			*B. malayi*	WHO [38]	2020	Targeted surveys, MX	Historic hotspots, migrants from endemic countries	2017 onwards
Togo	2017	AFR	*W. bancrofti*	Dorkenoo et al. [46]	2021	Targeted surveys, MX, MMDP, cat serosurveys	Migrants from endemic countries	2018
			*W. bancrofti*	Dorkenoo et al. [47]	2018	MX	Historic hotspots	2016–2017
Tonga	2017	WPR	*W. bancrofti*	Lawford et al. [48]	2025	Targeted surveys, health facility-based screening	Historic hotspots	2024
Egypt	2018	EMR	*W. bancrofti*	WHO [36]	2024	Unspecified PVS, MMDP	Unspecified	After 2018 (years unknown)
Palau	2018	WPR	*W. bancrofti*	WHO [49]	2020	Targeted surveys	Migrants from endemic countries	2017 onwards
Wallis and Futuna	2015	WPR	*W. bancrofti*	Couteaux et al. [50]	2025	Targeted surveys, health facility-based screening	Areas with greater prevalence of hypereosinophilia	2024
Kiribati	2019	WPR	*W. bancrofti*	WHO [51]	2023	Integrated surveillance	Unspecified	Unspecified
Yemen	2019	EMR	*W. bancrofti*	WHO [36]	2024	Unspecified PVS, MMDP	Unspecified	After 2019 (years unknown)
			*W. bancrofti*	WHO [52]	2022	Integrated surveillance and MX, MMDP	Unspecified	Unknown
Malawi	2020	AFR	*W. bancrofti*	Barrett et al. [53]	2024	MMDP	Unspecified	2020 onwards
Bangladesh	2023	SEAR	*W. bancrofti*	WHO [35]	2025	Targeted surveys, health facility-based screening	Historic hotspots, areas of low socioeconomic status	2023 onwards

* This column identifies whether priority populations were included in surveillance activities; however, sampling may not be limited to these groups. ^ Describes surveillance activities that took place at a provincial level after elimination within that province but prior to validation of elimination at a national level. WPR = WHO Western Pacific Region, SEAR = WHO South-East Asia Region, AFR = WHO African Region, EMR = WHO Eastern Mediterranean Region.

**Table 3:** Number of documents in which PVS activities were described, by country of activity.

WHO Region	Country	Year LF Eliminated	WHO-Recommended PVS Activities					Other Surveillance Activities		Sustained Surveillance ^	Alignment with 2025 WHO Guidelines (≥2 Strategies + Sustained Surveillance)
			Targeted Surveys	Integration into Existing Standardised Surveys	Health Facility-Based Screening	Molecular Xenomonitoring	Morbidity Management and Disability Prevention	Vector Surveys	Animal Reservoir Surveys		
AFR	Malawi	2020					1				
	Togo	2017	1			2	1				
AMR	Brazil *	2024									
EMR	Egypt	2018					1				
	Yemen	2019		1			1	1			
SEAR	Bangladesh	2023	1	1	1	1					
	Maldives	2016	3	2			1	1		✓	✓
	Sri Lanka	2016	8	1		5	2		2	✓	✓
	Thailand	2017	5	1		4	1		3	✓	✓
	Timor-Leste *	2024									
WPR	Cambodia	2016		1						✓	
	China	2007	5		1	2	3			✓	✓
	Cook Islands *	2016									
	Kiribati	2019		1							
	Lao PDR *	2023									
	RMI *	2017									
	Niue	2016		1							
	Palau	2018	1								
	South Korea	2008	2			4				✓	✓
	Tonga	2017	1		1						
	Vanuatu	2016		1							
	Vietnam *	2018									
	Wallis and Futuna	2018			1						
No. documents describing activities			28	10	4	18	11	2	5	-	-
No. countries in which activities occurred			9	9	4	6	8	2	2	-	-

Note: darker shading represents a greater number of documents in which activities were recorded. ✓ indicates that based on the activities observed in the included documents, these criteria were met. * Countries without PVS activities recorded in extracted literature, ^ Repeated PVS activities implemented over multiple years following validation, AFR = African Region, AMR = Region of the Americas, EMR = Eastern Mediterranean Region, SEAR = South-East Asian Region, WPR = Western Pacific Region, Lao PDR = Lao People’s Democratic Republic, RMI = Republic of the Marshall Islands.

**Table 4:** Number of documents in which health system constraints and enablers of PVS were described, by country of activity.

WHO Region	Country	Constraints					Enablers			
		Financing	Health Workforce and Technical Capacity	Accessing PVS Diagnostic Tools and Supplies	Policies and Guidelines	Policies and Guidelines	Health Workforce and Technical Capacity	Infrastructure Development	Data Development	Health Education
AFRO	Malawi	1	1							
	Togo	1	1	1						
SEAR	Bangladesh	1				1	1			
	Maldives		1	2		1			1	
	Sri Lanka	1	2	1	3	2	1		2	
	Thailand	3	1	2	1				1	1
WPRO	Cambodia	1	1	1						
	China				1	2		1	1	1
	South Korea					1	1		1	
	Tonga	1			1					
	Vanuatu	1	1	1						
No. of documents describing theme		10	9	8	6	7	3	1	6	2
No. of countries in which theme occurred		8	7	6	4	5	3	1	5	2

Note: darker shading represents a greater number of documents in which activities were recorded.

## Data Availability

The data presented in this study are available in PubMed at https://pubmed.ncbi.nlm.nih.gov/ (accessed on 2 September 2025), in Scopus at https://www.scopus.com/ (accessed on 2 September 2025), in Embase at https://www.embase.com/ (accessed on 2 September 2025), in Web of Science at https://www.webofscience.com/wos/woscc/basic-search (accessed on 2 September 2025), in WHO IRIS at https://iris.who.int/ (accessed on 2 September 2025).

## Supplementary Materials

The following supporting information can be downloaded at https://www.mdpi.com/article/10.3390/tropicalmed11010028/s1, File S1: Search terms by database.
