# Supplementary material for: Global Lymphatic Filariasis Post-Validation Surveillance Activities in 2025: A Scoping Review
Source: Trop Med Infect Dis. 2026 Jan 19;11(1):28. doi: 10.3390/tropicalmed11010028 (PMC12846383; doi:10.3390/tropicalmed11010028)
Supplement: Supplementary file 1 [file tropicalmed-11-00028-s001.zip › tropicalmed-4055749-supplementary.pdf]

File S1: Search terms by database

Database	Search terms
**PubMed**	("Bangladesh"[Mesh] OR Bangladesh* OR "Cambodia"[Mesh] OR Cambodia* OR Cook Island* OR "Egypt"[Mesh] OR Egypt* OR Kiribati* OR "I-Kiribati" OR "Laos"[Mesh] OR Laos OR "Malawi"[Mesh] OR Malawi* OR Maldiv* OR Marshall Island* OR Niue* OR Palau* OR "Sri Lanka"[Mesh] OR Sri Lanka* OR "Thailand"[Mesh] OR Thai* OR "Togo"[Mesh] OR Togo* OR Tonga* OR Vanuatu* OR "Vietnam"[Mesh] OR Vietnam* OR "Yemen"[Mesh] OR Yemen* OR "Wallis and Futuna" OR Wallis* OR Futuna* OR "Brazil"[Mesh] OR Brazil* OR "Timor-Leste"[Mesh] OR Timor*)<br>AND<br>("Elephantiasis, Filarial"[Mesh] OR "lymphatic filariasis" OR elephantias* OR filaria* OR "filarial elephantiasis" OR filarial lympho*dema OR "Wuchereria bancrofti" OR "Brugia malayi" OR "Brugia timori" OR Bancrofti* OR Brugia*)<br>AND<br>("Sentinel Surveillance"[Mesh] OR "sentinel surveillance" OR "Public Health Surveillance"[Mesh] OR "public health surveillance" OR "Population Surveillance"[Mesh] OR "population surveillance" OR "Monitoring, Physiologic"[Mesh] OR "physiologic monitoring" OR "Epidemiological Monitoring"[Mesh] OR "epidemiological monitoring" OR "Mass Screening"[Mesh] OR "mass screening")<br>AND<br>("post-elimination" OR "post-validation" OR elimination OR validation)<br>AND<br>(PUBYEAR > 2006 AND PUBYEAR < 2026)
**Scopus**	(Bangladesh* OR Cambodia* OR Cook Island* OR Egypt* OR Kiribati* OR “I-Kiribati” OR Lao* OR Malawi* OR Maldiv* OR Marshall Island* OR Niue* OR Palau* OR Sri Lanka* OR Thai* OR Togo* OR Tonga* OR Vanuatu* OR Vietnam* OR Yemen* OR Wallis* OR Futuna* OR Brazil* OR Timor*)<br>AND<br>("lymphatic filariasis" OR "elephantiasis" OR "filarial elephantiasis" OR "Wuchereria bancrofti" OR "Brugia malayi" OR "Brugia timori" OR "filarial infection”)<br>AND<br>("sentinel surveillance" OR "public health surveillance" OR "population surveillance" OR "physiologic monitoring" OR "epidemiological monitoring" OR "mass screening")<br>AND<br>("post-elimination" OR "post-validation" OR elimination OR validation)<br>AND<br>(PUBYEAR > 2006 AND PUBYEAR < 2026)
**Embase**	(Bangladesh* OR Cambodia* OR Cook Island* OR Egypt* OR Kiribati* OR “I-Kiribati” OR Lao* OR Malawi* OR Maldiv* OR Marshall Island* OR Niue* OR Palau* OR Sri Lanka* OR Thai* OR Togo* OR Tonga* OR Vanuatu* OR Vietnam* OR Yemen* OR Wallis* OR Futuna* OR Brazil* OR Timor*)<br>AND<br>("lymphatic filariasis" OR "elephantiasis" OR "filarial elephantiasis" OR "Wuchereria bancrofti" OR "Brugia malayi" OR "Brugia timori" OR "filarial infection”)<br>AND<br>("sentinel surveillance" OR "public health surveillance" OR "population surveillance" OR "physiologic monitoring" OR "epidemiological monitoring" OR "mass screening")

Database	Search terms
	AND<br>("post-elimination" OR "post-validation" OR elimination OR validation)<br>AND<br>[2007-2025]/py
**Web of Science**	(Bangladesh* OR Cambodia* OR Cook Island* OR Egypt* OR Kiribati* OR “I-Kiribati” OR Lao* OR Malawi* OR Maldiv* OR Marshall Island* OR Niue* OR Palau* OR Sri Lanka* OR Thai* OR Togo* OR Tonga* OR Vanuatu* OR Vietnam* OR Yemen* OR Wallis* OR Futuna* OR Brazil* OR Timor*)<br>AND<br>("lymphatic filariasis" OR "elephantiasis" OR "filarial elephantiasis" OR "Wuchereria bancrofti" OR "Brugia malayi" OR "Brugia timori" OR "filarial infection”)<br>AND<br>("sentinel surveillance" OR "public health surveillance" OR "population surveillance" OR "physiologic monitoring" OR "epidemiological monitoring" OR "mass screening")<br>AND<br>("post-elimination" OR "post-validation" OR elimination OR validation)<br>AND<br>[2007-2025]/py
**WHO IRIS**	(Bangladesh* OR Cambodia* OR Cook Island* OR Egypt* OR Kiribati* OR “I-Kiribati” OR Lao* OR Malawi* OR Maldiv* OR Marshall Island* OR Niue* OR Palau* OR Sri Lanka* OR Thai* OR Togo* OR Tonga* OR Vanuatu* OR Vietnam* OR Yemen* OR Wallis* OR Futuna* OR Brazil* OR Timor*)<br>AND<br>("lymphatic filariasis" OR "elephantiasis" OR "filarial elephantiasis" OR "Wuchereria bancrofti" OR "Brugia malayi" OR "Brugia timori" OR "filarial infection”)<br>AND<br>("sentinel surveillance" OR "public health surveillance" OR "population surveillance" OR "physiologic monitoring" OR "epidemiological monitoring" OR "mass screening")<br>AND<br>("post-elimination" OR "post-validation") [1]<br>AND<br>Date issued: [2007 TO 2025]

[1] The terms ‘validation’ and ‘post-validation’ refer to distinct stages of the elimination process in WHO publications; the terms ‘elimination’ and ‘validation’ were removed when searching this database to maintain specificity.
